# Complete coding genome sequence of lettuce necrotic yellows virus: a case study of garlic biosecurity status

**DOI:** 10.1128/mra.00510-26

**Published:** 2026-06-22

**Authors:** Solomon Maina, Leonard Tesoriero

**Affiliations:** 1 Elizabeth Macarthur Agricultural Institute, New South Wales Department of Primary Industries and Regional Development (DPIRD) 153388, Menangle, NSW, Australia; Portland State University, Portland, Oregon, USA

**Keywords:** virus, genomes, biosecurity, crops, garlic

## Abstract

We report the genome sequence of lettuce necrotic yellows virus isolate LY from garlic (*Allium sativum*) collected in New South Wales, Australia (1980), showing 3′-N-P-4b-M-G-L-5′ organization. It shares 97.8% identity with a New Zealand isolate and 81.28% with another Australian isolate, underscoring post-entry quarantine for imported garlic germplasm biosecurity.

## ANNOUNCEMENT

Lettuce necrotic yellows virus (LNYV) (*Alphacytorhabdovirus lactucanecante*) is the type species of the genus *Cytorhabdovirus* (1). LNYV causes serious disease in lettuce (*Lactuca sativa* L.) and is transmitted in a persistent, propagative manner by the aphid *Hyperomyzus lactucae* (2–4). LNYV genome consists of a monopartite, negative-sense, single-stranded RNA of about 13,000 nucleotides (nts) (3).

Isolate LY (Aus_Ly_80) was collected in 1980 from a garlic (*Allium sativum*) plant in Yanco, New South Wales, Australia, and has since been maintained at the NSW Plant Pathology and Mycology Herbarium under accession number 35868. The dried leaf was subjected to RNA extraction using a Zymo Plant RNA Miniprep Kit, and a library was prepared using the TruSeq Stranded total RNA with Ribo-Zero Plant (Illumina) (5–7). The library was diluted, denatured, and then sequenced using a MiSeq v3 kit (Illumina) with paired-end reads (2 × 281 cycles). Quality control of the raw read fastq files was performed using CLC Genomics Workbench version 25 (Qiagen), with quality scores limit set to 0.01, maximum number of ambiguities to 2, and removing any reads with <50 nt. *De novo* assembly was performed using CLCGW with the following settings: automatic word size, automatic bubble size, minimum contig length of 800 bp, mismatch cost of 2, insertion cost of 3, deletion cost of 3, length fraction of 0.5, and similarity fraction of 0.9 (6, 7). The contigs of interest were imported into Geneious Prime (version 2026.0.2, Biomatters, New Zealand), multiple alignment with GenBank sequences was derived using MUSCLE 5.1 available in Geneious, and annotations were performed using transfer annotation with the similarity threshold set to 80%, while other settings were left as default (5).

High-throughput sequencing (HTS) generated 2,099,304 raw reads, of which 2,093,887 reads remained after quality filtering. *De novo* assembly produced 372 contigs, and BLASTn analysis of assembled contigs using BLAST+ v2.7 (8) identified a contig corresponding to the LNYV genome designated L6A-LY (GenBank accession LC897112.1). This contig was 12,769 nt in length, with 155,377 reads mapping to it, an average coverage depth of 251×, and a GC content of 43%. The L6A-LY shared 97.8% nt identity with the New Zealand isolate NGSTPS18/HZ17-73 (MW848533) and 81.28% nucleotide identity with an isolate (NC_007642) previously recovered from a garlic plant in Victoria, Australia. The genome structure was typical of LNYV, comprising the 3′-N-P-4b-M-G-L-5′, where N is the nucleocapsid gene, P is the putative phosphoprotein gene, 4b encodes an unknown protein function, M is the matrix protein gene, G is the glycoprotein gene, and L is the polymerase gene (9).

Globally, numerous viruses infecting garlic have been characterized, and their impacts on crop yield have been well documented (10–13). In contrast, information on the distribution and impact of garlic viruses in Australia remains limited (14–16). The Australian garlic industry relies on imported germplasm to meet market demand and supply of planting material (17). Such repeated introductions increase the likelihood of introducing novel or genetically divergent virus species (18, 19). A robust post-entry quarantine (PEQ) framework for garlic germplasm, integrating HTS into PEQ screening, would enable accurate assessment of viral risks associated with imported garlic material. This will mitigate biosecurity risks while facilitating the distribution of clean, high-quality propagules to commercial growers to enhance productivity within the Australian garlic industry.

## Data Availability

The sequence was deposited in GenBank under accession number LC897112.1. The raw reads are available in SRA under SRR36616580 and BioProject number PRJNA1394657.
